# Interleukin-1β rs16944 and rs1143627 polymorphisms and risk of developing major depressive disorder: A case-control study among Bangladeshi population

**DOI:** 10.1371/journal.pone.0317665

**Published:** 2025-01-22

**Authors:** Faria Mehreen Toma, Khondoker Tashya Kalam, Md. Aminul Haque, Sejuti Reza, Raushanara Akter, Mohammad Safiqul Islam, Md. Rabiul Islam, Zabun Nahar

**Affiliations:** 1 Department of Pharmacy, University of Asia Pacific, Dhaka, Bangladesh; 2 School of Pharmacy, BRAC University, Dhaka, Bangladesh; 3 Department of Pharmacy, Noakhali Science and Technology University, Noakhali, Bangladesh; Fundación Universitaria del Área Andina, COLOMBIA

## Abstract

**Background:**

Epidemiological research suggests that altered levels of cytokine are associated with pathophysiology and the development of major depressive disorder (MDD). Based on earlier study, IL-1β rs16944 and rs1143627 polymorphisms may increase the risk of depression. Here, we aimed to evaluate the correlation between these polymorphisms and MDD susceptibility among the population in Bangladesh.

**Methods:**

Blood samples were collected from 100 MDD patients and 70 matched controls. Study participants were evaluated by DSM-5 criteria and PCR-RFLP method were applied for genotyping.

**Results:**

The IL1β rs1143627 and rs16944 polymorphisms were found to have a significant association with the risk of MDD. In case of rs1143627 CT heterozygous genotype (OR = 2.22, 95% CI = 1.08–4.55, p-value = 0.029) and combined CT+TT (OR = 2.35, 95% CI = 1.15–4.79, p-value = 0.019) genotype was strongly associated with the increased risk of MDD in comparison to CC common genotype. Moreover, the over-dominant model indicated a 2.15-fold higher risk for MDD development (OR = 2.15, 95% CI = 1.05–4.40, p-value = 0.036). On the other hand, the IL1β rs16944 polymorphisms revealed that the TC+CC combined genotype in the dominant model showed a 2.06-fold increased risk for MDD development compared to the TT common homozygote (OR = 2.06, 95% CI = 1.06–3.99, p-value = 0.032).

**Conclusion:**

Studies suggests that IL1β rs16944 and rs1143627 polymorphisms are associated with an increased risk of MDD. These findings will provide us with valuable insights into the pathophysiology of MDD.

## 1. Introduction

Major depressive disorder (MDD) is the most common disabling psychiatric disorder characterized by sadness, anhedonia, apathy, irritability, loss of motivation, poor attention and decision-making, behavioral despair, cognitive abnormalities, and avolition [1–3]. The WHO estimated a substantial global burden of mental health disorders in 2019; among them, 264 million people experienced depression, and 45 million had bipolar disorder. About 50 million individuals had dementia, and 20 million had schizophrenia and other psychoses. These data demonstrate the significant impact of mental health among the population worldwide. Intellectual disability and autism spectrum disorders are common neurodevelopmental issues that develop in children and teens [4]. It ranks as the second-highest cause of global morbidity, posing a considerable public health concern [5]. Patients with MDD have a nearly 20-fold increased risk of suicide compared to the general population [6]. After puberty, women have a two-fold higher risk of having MDD than men. Women tend to experience episodes more frequently than men, rather than longer episodes with different treatment responses or higher recurrence rates [1, 7].

MDD is a complex disorder with multiple etiologies. While the exact reasons for mental diseases often remain unknown, theories combine various hypotheses. Understanding these conditions involves behavior, feelings, perceptions, and thoughts, with psychiatric disorders requiring consideration of societal standards, cultural values, and religious practices [4]. Many studies have demonstrated numerous complexes and interrelated molecular pathways underpinning MDD. The pathways or systems under consideration include stress, inflammation, monoamines, excitatory and inhibitory neurotransmission, genetics, epigenetics, environmental factors, neurotrophins and neurogenesis, the opioid system, mitochondrial dysfunction, myelination, the gut-brain axis, the hypothalamic-pituitary (HPA) axis, etc. [8]. Hyperactivity of the HPA axis, impaired neurogenesis, and decreased hippocampal volumes caused by glucocorticoid receptor malfunction are contributing factors to MDD. Neurotrophins are growth factors involved in the generation, support, and plasticity of neuronal networks, and brain-derived neurotrophic factor (BDNF) is a neurotrophins member that belongs to the neurotrophins family that activates tropomyosin- related kinase (Trk) and p75 receptors [8]. Reduced neurotrophic growth, indicated by low levels of BDNF, is a significant cause that leads to MDD [9]. Decreased levels of monoamines, especially 5- hydroxy-tryptamine (5-HT), noradrenaline, and dopamine, indicates the underlying mechanisms associated with MDD [8]. Findings from the blood and cerebrospinal fluid (CSF) analysis of MDD patients demonstrated enhanced levels of proinflammatory cytokines, inflammatory cytokines, chemokines, and soluble adhesion molecules. In addition, MDD patients also had higher levels of tumor necrosis factor-alpha (TNF-*α*) compared to healthy individuals [4]. Another study reported increased levels of serum IL-3 and an abatement of lipocalin-2 concentration in MDD patients [10]. Menezes Galvão *et al*. reported an elevated concentration of serum cortisol (SC) and serum salivary cortisol awakening response (CAR) in MDD patients compared to healthy controls (HCs) [11].

Several types of research evaluating the connection between genetic polymorphism and MDD exhibit a great interest in determining specific genes as targeted therapies to prevent and treat this extremely disabling disorder. A significant number of studies have demonstrated the involvement of different genetic polymorphisms in MDD. It is noteworthy to mention that a significant association of six genes with MDD susceptibility has been revealed by meta-analyses of genetic studies, and the identified genes include *APOE-SLC6A4-*apolipoprotein E, SLC6A3- dopamine transporter and SLC6A4-serotonin transporter, *DRD4-*dopamine receptor D4, MTHFR-methylenetetrahydrofolate reductase, and GNB3-guanine nucleotide-binding protein [12]. It has been demonstrated that a 44-bp repeat polymorphism in the gene’s promoter region (5-HTTLPR) affects the serotonin transporter’s expression levels *in vitro*, making this functional variant an evident candidate for further research of MDD [13–15]. A small increase in the risk of MDD is attributed to the S allele of the 5-HTTLPR gene [16]. A correlation of the T allele of the C825T polymorphism in GNB3 with MDD was identified in a study conducted on the Korean population [17, 18]. A link between the -1438A/G polymorphism of the promoter region of the 5- HTR2A gene and MDD has also been established by a previous study conducted on the same population [19, 20]. Another investigation taking 91 SNPs in 21 candidate genes demonstrated a nominally significant association of SNPs in HTR2C, CCKAR, DRD1, and DRD2 genes with MDD [21].

Interleukin-1 beta (IL-1β), a crucial proinflammatory cytokine produced by the IL-1β gene, is linked to persistent inflammation and holds a significant role in inflammatory conditions. Increased concentrations of IL-1 proteins, especially IL-1β, significantly amplify the strength of the inflammatory reaction [22]. The role of IL-1 in MDD has been studied extensively across various investigations. IL-1 is thought to be associated with MDD diagnosis, symptoms, and antidepressant response [23]. Additionally, the IL-1 cytokine generated by the IL-1β gene may be useful in pharmacogenetic investigations. Important cellular processes involved such as proliferation, differentiation, and apoptosis, plays a significant role in orchestrating the inflammatory response. Selecting and genotyping IL-1 variants may provide important information [24]. IL-1β mediates communication between the immunological and central nervous systems. Elevated IL-1 levels are associated with mood spectrum difficulties, which are regulated by genetic alterations in the IL-1β gene under distress [25]. The IL-1β gene is situated at locus 2q14.1 on chromosome 2. Numerous SNPs have been documented for this gene, and among them, rs1143627 and rs16944 have been linked to MDD. The rs1143627 polymorphism is located in the promoter region of the IL-1β gene. This region plays a crucial role in regulating gene expression and determining the amount of IL-1β protein produced. Certain genetic variants at this location might influence the binding of transcription factors, leading to changes in IL-1β gene expression. Consequently, individuals with different genotypes at rs1143627 might produce varying levels of IL-1β [26]. A number of remarkable studies have investigated the association of IL-1β gene rs16944 polymorphisms with both MDD susceptibility and its treatment efficacy [27]. A case-control study was done that compared individuals with MDD and observed variation in the L1B rs16944 polymorphism associated with antidepressant medication response [28]. Another study suspected that a genetic alteration in the IL1B gene influenced the alleviation rate in MDD patients after antidepressant treatment [29]. Researchers investigated the association between rs1143627 polymorphism and different diseases to understand its potential role in disease susceptibility, severity, and therapeutic outcomes. In case of MDD, scientists investigate whether individuals carrying specific genotypes of rs1143627 have an altered risk of developing depression or experience varying levels of depressive symptoms. Furthermore, alignment with IL1-β and IL-6R polymorphisms and its therapeutic outcomes in severe depression have been expressed [30].

All these findings suggest that polymorphisms in the IL1-β gene play an important role in MDD and may be a factor in the risk of developing MDD. Therefore, this study aimed to examine the potential association of IL1-β rs16944 and rs1143627 with MDD.

## 2. Materials & method

### 2.1 Materials

Emerald Amp GT PCR Master Mix, DNA ladder and restriction enzymes were obtained from Takara Bio Inc (Japan). Agarose and TAE buffer were bought from Sigma Life Science (USA). DNA extraction kit was purchased from Favorgen (USA). Ethidium bromide was taken from Alfa Aesar (UK). Primers were from GCC Biotech (India).

### 2.2 Subjects

Complete physical, neurological, and laboratory examinations were performed to demonstrate that all patients were free of significant physical and (or) neurological illnesses. All patients and HCs were from Dhaka, Bangladesh. We assumed the exposed controls and alpha risks as 20% and 5%, respectively. Also, this case-control study expected an odds ratio of 3 with statistical power of 80%. Based on above assumptions, the estimated sample size was supposed to be 174 and we enrolled 100 MDD patients and 70 control subjects. The patients were recruited from the Department of Psychiatry of Bangabandhu Sheikh Mujib Medical University (BSMMU), Dhaka, Bangladesh. Both the inpatient and outpatient sections were adequately set up. A qualified psychiatrist diagnosed MDD patients and HCs according to the diagnostic and statistical manual for mental disorders, 5^th^ edition (DSM-5). The severity of MDD patients was assessed using the Hamilton depression rating scale (Ham-D). Both MDD patients and HCs were matched by their age and sex. The present study excluded subjects with chronic illness, psychiatric comorbidities, pregnancy, and addiction of alcohol or any other substances. We also excluded subjects who are taking any antidepressant or antipsychotic medications for at least two preceding weeks. Before data and sample collection, we briefed each subject about the objective and purpose of study and obtained informed written consent for their participation. In the case of individuals with impaired decision-making capacity, we obtained similar informed written consent from their legal guardians. The genotyping analysis was carried out in the Biotechnology Research Lab, Department of Pharmacy, University of Asia Pacific, Bangladesh.

### 2.3 DNA extraction and genotyping

Blood samples from all participants were collected between March 1, 2023, and August 30, 2023. During sample collection, each tube contained EDTA and then the blood sample was collected in the following labelled tubes. Genomic DNA for the SNP study was extracted using a commercial genomic DNA extraction kit (Favorgen, USA) by following their provided protocol. In brief, the blood samples were lysed and transferred to binding column tubes. After washing with different buffers, the DNA was eluted using nuclease-free water and stored at -20°C. The purity of the isolated DNA was checked using 1% agarose gel, and quantification was performed at 260 nm using a UV-VIS spectrophotometer (Shimadzu Corporation, Japan). Polymerase chain reaction-restriction fragment length polymorphism (PCR-RFLP) assay was employed to identify single nucleotide polymorphisms (SNPs) at different genomic sites. A summary of the thermal conditions and primer details utilized for amplifying the target regions is provided in Table 1. The PCR (Thermal Cycler, Exco Healthcare, Singapore) was conducted using Emerald Amp GT PCR Master Mix, and confirmation was achieved through 1% agarose gel electrophoresis upon completion. Following PCR, products were subjected to digestion using Alu I (at 37°C for 16 hours) and Ava I (at 37°C for 6 hours) restriction enzymes to examine the SNP at IL-1β rs1143627 and rs16944 respectively. Visualization of the digested products was performed via gel electrophoresis using ethidium bromide-containing 2% agarose. After PCR, all amplified products were analyzed using agarose gel electrophoresis to assess their purity. The presence of a single band confirmed the purity of the amplified products, ensuring the reliability of the results obtained after digestion. If multiple bands were observed in any sample after PCR, it was excluded from the restriction digestion reaction. Additionally, duplicate testing was carried out on a subset of samples (20% of the total population) to validate the obtained results and to ensure quality, restricted fragments with faint or low-intensity bands within the same group were excluded from the analysis.

**Table 1:** Primer sequences, thermal conditions and amplified products’ size.

Gene and Enzyme	Primers Sequence	Thermal Condition	Fragments, BP
	F-Forward, R-Reverse		
IL-1βrs1143627 and Alu I	F: 5’-AGAAGCTTCC ACCAATACTC-3’	3 min of denaturation at 94°C; 35 cycles of 94°C for 30 sec, 54°C for 30 sec, 72°C for 45 sec. The final extension was at 72°C for 10 min.	PCR product: 239 bp CC Common homozygote (5bp and 234bp) CT Heterozygote (5bp, 97bp, 137bp and 234bp) TT Rare homozygote (5bp, 97bp and 137bp)
	R: 5’-ACCACCTAGT TGTAAGGA-3’		
IL-1β rs16944 and Ava I	F 5’-TGGCATTGAT CTGGTTCATC-3’	4 min of denaturation at 94°C; 30 cycles of 94°C for 45 sec, 57°C for 30 sec, 72°C for 45 sec. The final extension was at 72°C for 10 min.	PCR product: 305 bp TT Common homozygote (305bp) TC Heterozygote (111bp, 194bp & 305bp) CC Rare homozygote (111bp & 194bp)
	R 5’-GTTTAGGAATCTTCCCACTT-3’		

***Note*: *Components of PCR reaction*:** *PCR 2X master mix (10 μL)*, *DNA (2 μL of 100ngm/ μL)*, *forward primer (2 μL of 5 pmole/ μL)*, *reverse primer (2 μL of 5 pmole/ μL)*, *ddH2O (4 μL)*. ***Components of Restriction digestion reaction*:** *PCR amplified product (8 μL)*, *10X QuickCut buffer (1 μL)*, *and enzyme (1μL)*.

### 2.4 Statistical analyses

Demographic data, clinical data, and genomic frequency were compared between cases and controls with a Chi-square (χ2) test and unpaired t-tests. Multivariate logistic regression was used to assess the odds ratios (ORs) with 95% confidence intervals (CIs). ORs were adjusted using different covariates, such as age, gender, and BMI. All statistical analyses were performed by applying the SPSS software, version 17.0. Also, Bonferroni adjustments were used to account for multiple comparisons.

### 2.5 Ethical consideration

The research protocol was approved by the Research Ethics Committee of University of Asia Pacific, Dhaka, Bangladesh (Ref: UAP/REC/2023/108). The objectives of the study were communicated to the participants, and written consent was obtained from each participant. We conducted this investigation in accordance with the Helsinki Declaration’s guiding principles.

## 3. Results

### 3.1 Socio-demographic history

The socio-demographic characteristics of 100 MDD patients and 70 HCs are summarized in Table 2. Among the MDD patients, 35% were aged between18-25, 32% were aged between 26–35, 25% aged between were 36–45, and 8% were aged between 46–60. Similar age distribution was observed in HCs, with 35% aged between 18–25 years old. The ages of both groups ranged from 18 to 60 years, with an average age of 31.69 for patients and 31.34 for controls (p-value = 0.998). The average BMI was 23.51 kg/m^2^ for patients and 24.64 kg/m^2^ for controls. In both groups, 70% were married, and 50% lived in urban areas. P-values for economic status, occupation, and education level were 0.996, 1.000, and 0.808, respectively. Among the patients, 54% had a previous history of MDD, and 30% had a family history of the disorder, with 46 being newly diagnosed. The p-values for DSM-5 and Ham-D scales were both < 0.001, confirming MDD diagnosis.

**Table 2:** Socio-demographic profile of study participants.

Characteristics	MDD patients (n = 100) Mean ± SEM	Healthy controls (n = 70) Mean ± SEM	p-value
**Age in years**	31.69±0.996	31.34±1.196	0.998
18–25	35 (35%)	25 (35%)	
26–35	32 (32%)	22 (32%)	
36–45	25 (25%)	17 (25%)	
46–60	8 (8%)	6 (8%)	
**Sex**			0.984
Male	27 (27%)	19 (27%)	
Female	73 (73%)	51 (73%)	
**Marital status**			1.000
Married	70 (70%)	49 (70%)	
Unmarried	30 (30%)	21 (30%)	
**BMI (kg/m** ^**2**^	23.51±0.49	24.64±0.42	0.134
18.5–25.0 (Normal)	54 (54%)	32 (46%)	
Above 25.0 (Obese)	33 (33%)	33 (47%)	
Below 18.5 (CED)	13 (13%)	5 (7%)	
**Education level**			0.808
Illiterate	4 (4%)	1(4%)	
Primary	15 (15%)	11 (15%)	
Secondary	40 (40%)	28 (40%)	
Graduate and above	41 (41%)	30 (41%)	
**Occupation**			1.000
Business	5 (5%)	4 (5%)	
Service	16 (16%)	11(16%)	
Unemployment	24 (24%)	17 (24%)	
Student	6 (6%)	4 (6%)	
Housewife	45 (45%)	31(45%)	
Others	4 (5%)	3(5%)	
**Economic impression**			0.996
Low	19 (19%)	13(19%)	
Lower middle	60 (60%)	42 (60%)	
Upper middle	21 (12%)	15 (66%)	
**DSM-5**	7.36±0.134	0.81±0.119	**<0.001**
**Ham-D score**	18.50±4.72	0.86±0.172	**< 0.001**
**Smoking habit**			**0.039**
Yes	8 (8%)	13 (18%)	
No	92 (92%)	57 (82%)	
**Residence area**			0.702
Rural	50 (50%)	35 (50%)	
Urban	50 (50%)	35 (50%)	
**Previous history of MDD**			**< 0.001**
Yes	54 (54%)	0	
No	46 (46%)	70 (100%)	
**Family history of MDD**			**< 0.001**
Yes	30 (30%)	0	
No	70 (70%)	70 (100%)	

Abbreviations: BMI, body mass index; CED, chronic energy deficiency; Ham-D, Hamilton depression rating scale; MDD, major depressive disorder; SEM, standard error mean. Significant p-values are presented in bold.

### 3.2 Association of MDD with IL-1β rs1143627 polymorphism

Based on variations in chromosome bases and fragment sizes, three types of genetic variations were identified among the participants: CC representing the common homozygote (where both chromosomes carry the same allele, H = C), CT representing the heterozygote (where one chromosome carries allele G while the other carries allele H), and TT representing the rare homozygote (where both chromosomes carry allele T). Analysis of genotypes and allelic frequencies of the IL-1β rs1143627 polymorphism in cases revealed that 63% of patients exhibited the CC common homozygote genotype, 35% had the CT heterozygote genotype, and there was 2% TT rare homozygote genotype. The distribution of genotypes among patients adhered to Hardy-Weinberg equilibrium (HWE) (p-value> 0.05) (χ2 = 1.32, p-value = 0.250), indicating a consistent genotypic distribution among individuals with MDD (Table 3). Upon analysis, it was noted that individuals with the CT heterozygote genotype exhibited a 2.22 times higher risk of developing MDD compared to those with the CC common homozygote genotype (CT vs. CC: OR = 2.22, 95% CI = 1.08–4.55, p-value = 0.029), revealing that the association reached statistically significant value. Likewise, individuals with the TT rare homozygote genotype demonstrated a 4.44 times higher risk of developing MDD compared to those with the CC common homozygote genotype, and a statistically insignificant association was observed (TT vs. CC: OR = 4.44, 95% CI = 0.21–94.64, p-value = 0.338). When considering the combined genotype CT+TT in the dominant model, there was a 3.15 times increased risk for MDD development compared to the CC genotype and this association showed statistically significant results (Dominant model—CT+TT vs. CC: OR = 2.35, 95% CI = 1.15–4.79, p-value = 0.019). In the recessive model, the CC+CT combined genotype showed a 3.58 times greater risk for MDD compared to the TT genotypes, yet no statistically significant results were observed (Recessive model—TT vs. CC+CT: OR = 3.58, 95% CI = 0.17–75.70, p-value = 0.413). Moreover, the over-dominant model indicated a 2.15-fold higher risk for MDD development. This change was statistically significant (Over-dominant model—CT vs. CC+TT: OR = 2.15, 95% CI = 1.05–4.40, p-value = 0.036). The result of allele also showed that C allele might be a risk factor for the development of MDD (OR = 2.25, 95% CI = 0.99–5.09, p-value = 0.052).

**Table 3:** Association between major depressive disorders with IL1β rs1143627 polymorphism.

Allele	MDD patients (n)	HWE		Healthy controls (n)	HWE		Genetic models	OR	95% CI	*p-value
		χ^2^	*p*		χ^2^	*p*				
CC	63	1.32	0.250	56	0.86	0.352	Additive model 1 (CT vs. CC)	2.22	1.08–4.55	**0.029**
Common homozygote (5bp and 234bp)										
							Additive model 2 (TT vs. CC)	4.44	0.21–94.64	0.338
CT	35			14			Dominant model CT+TT vs. CC	2.35	1.15–4.79	**0.019**
Heterozygote (5bp, 97bp, 137bp and 234bp)										
TT	2			0			Recessive model TT vs. CC+CT	3.58	0.17–75.70	0.413
Rare homozygote (5bp, 97bp and 137bp)										
							Over dominant model	2.15	1.05–4.40	**0.036**
							CT vs CC+TT			
							C vs T	2.25	0.99–5.09	**0.052**

Abbreviations: CI, confidence interval; HWE, Hardy-Weinberg equilibrium; MDD, major depressive disorder; OR, odds ratio. p-value>0.05 obeyed Hardy- Weinberg equilibrium. A p-value of less than 0.05 indicates statistical significance. Significant p-values are presented in bold.

*Bonferroni adjusted p values for multiple comparisons.

### 3.3 Association of MDD with IL-1β rs16944 polymorphism

**Fig 1:**
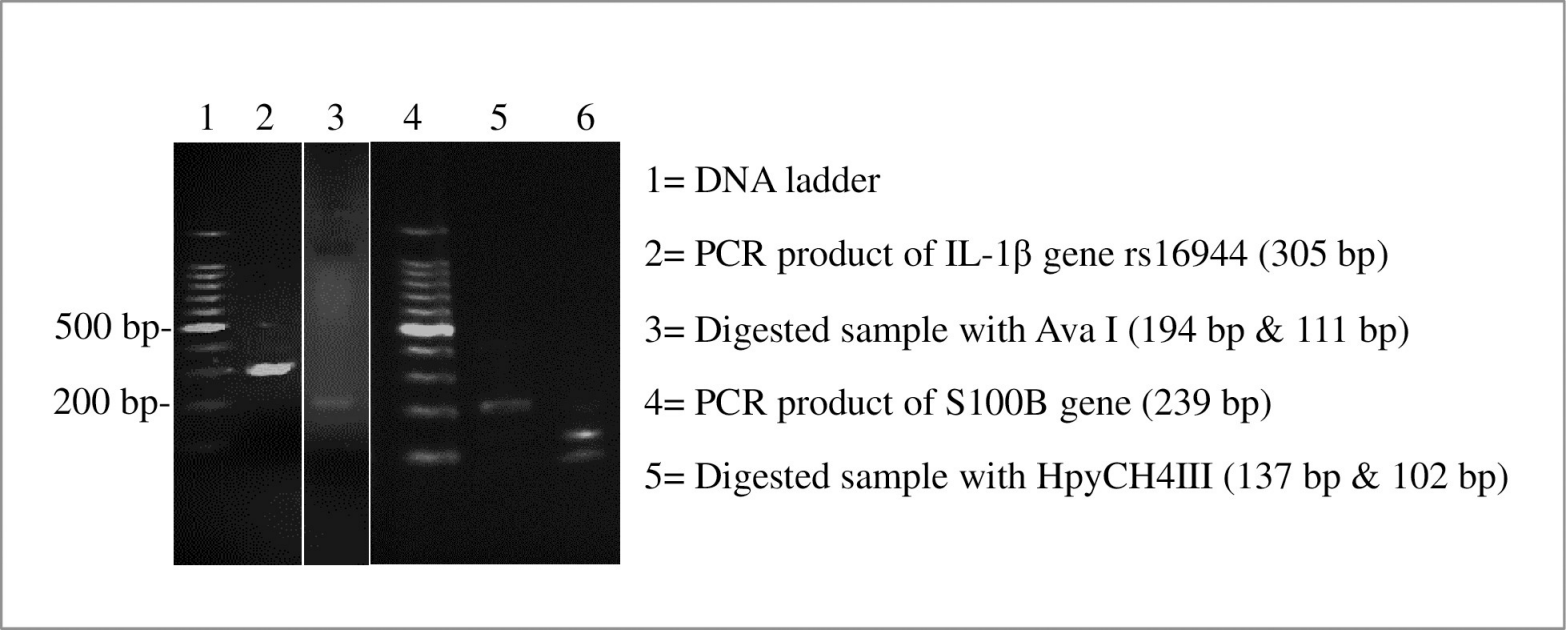
Representative data of agarose gel electrophoresis.

The association between IL-1β rs16944 polymorphism with MDD is shown in Table 4. Analysis of genotypes and allelic frequencies of the IL-1β gene rs16944 polymorphism in studies revealed that 56% of patients exhibited the TT common homozygote genotype, 40% had the TC heterozygote genotype, and 4% had the CC rare homozygote genotype. The genotype distributions of the patients adhered to the Hardy-Weinberg equilibrium, with a p-value of 0.334 (p-value > 0.05) in the cases (χ2 = 0.93, p-value = 0.334). Thus, it is evident from the Hardy-Weinberg equilibrium that there was a consistent genotypic distribution among individuals with MDD. Upon analysis, it is noted that individuals with the TC heterozygote genotype exhibit a 1.91 times increased risk of developing MDD compared to those with the TT common homozygote genotype (TC vs. TT: OR = 1.91, 95% CI = 0.99–3.73, p-value = 0.055), where the genotypic distribution was not statistically significant although it was very close. Similarly, individuals with the CC rare homozygote genotype also demonstrate an 8.20 times greater risk for MDD compared to those with the TT common homozygote genotype, yet this association was statistically insignificant (CC vs. TT: OR = 8.20, 95% CI = 0.43–156.12, p-value = 0.161). TC+CC combined genotype in the dominant model showed 2.06 times increased risk for MDD development when compared with the TT genotype and the association was significant (Dominant model—TC+CC vs. TT: OR = 2.06, 95% CI = 1,06–3.99, p-value = 0.032). In the recessive model, 6.57 times greater risk for MDD was demonstrated (Recessive model—CC vs. TT+TC: OR = 6.58, 95% CI = 0.35–124.11, p-value = 0.209) with no significant results. The over-dominant model revealed 1.79-fold greater risk for the development of MDD and the change was not statistically prominent (over-dominant model—TC vs. TT+CC: OR = 1.79, 95% CI = 0.92–3.47, p-value = 0.084). The allele distribution showed that T allele might be also a risk factor (OR = 1.94, 95% CI = 0.94–4.01, p-value = 0.074). A representative image of agarose gel electrophoresis has been presented in Fig 1.

**Table 4:** Association between major depressive disorders with IL1β rs16944 polymorphism.

Allele	MDD patients (n)	HWE		Healthy controls (n)	HWE		Genetic models	OR	95% CI	*p-value
		χ^2^	*p*		χ^2^	*p*				
TT	56	0.93	0.334	51	1.72	0.188	Additive model 1 (TC vs. TT)	1.91	0.99–3.73	**0.055**
Common homozygote (305bp)										
							Additive model 2 (CC vs. TT)	8.20	0.43–156.12	0.161
TC	40			19			Dominant model (TC+CC vs. TT)	2.06	1.06–3.99	**0.032**
Heterozygote										
(111bp, 194bp and 305bp)										
CC	4			0			Recessive model (CC vs. TT+TC)	6.57	0.35–124.11	0.209
Rare Homozygote (111bp & 194bp)										
							Over dominant model	1.79	0.92–3.47	0.084
							TC vs TT+TC			
							T vs C	1.94	0.94–4.01	0.074

Abbreviations: CI, confidence interval; HWE, Hardy-Weinberg equilibrium; MDD, major depressive disorder; OR, odds ratio. p-value>0.05 obeyed Hardy- Weinberg equilibrium; A p-value of less than 0.05 indicates statistical significance. Significant p-values are presented in bold.

*Bonferroni adjusted p values for multiple comparisons.

## 4. Discussion

MDD is a severe mental illness that worsens quality of life and increases the risk of disability [31]. While its pathophysiology is complex and not fully understood, MDD often runs in families, with first-degree relatives of depressed individuals are at a higher risk [19]. In our study, no socio-demographic factors were found to be statistically significant as risk factors for MDD in the Bangladeshi population (p-value > 0.05). Also, there were no possible association was observed between IL-1β rs1143627 (χ2 = 1.32, p-value = 0.250) and IL1β rs16944 (χ2 = 0.930, p-value = 0.334) gene polymorphisms and MDD.

The rs1143627 (-31 C>T) polymorphism in IL1β is being studied as a potential regulator of its expression. A T>C change at position -31 may disrupt the TATA box [32], affecting IL-1β production. Some studies have shown increased IL-1β expression in cells with the -31 T allele compared to the wild-type -31 C allele [33]. However, the findings are inconsistent, and some studies did not identify a clear link between SNP rs1143627 and IL-1β production in lab settings. Conversely, other research suggests that the C allele is associated with higher IL-1β expression in living organisms [34]. Another study observed that polymorphisms of rs1143627 and rs16944 influence IL-1β expression [35]. Researchers are exploring whether this polymorphism influences the risk or severity of MDD. Moreover, research on candidate genes and examine the impact of I IL-1β variations on psychiatric morbidity identified connections with various psychiatric disorders [36]. The examination of haplotypes involving SNP rs1143627 revealed significant distinctions in the variants of this polymorphism among individuals with major recurrent depression compared to the control group [37]. Additionally, rs1143627 was associated with schizophrenia and bipolar disorder, along with implications for whole-brain grey matter deficits in individuals with bipolar conditions [38].

Interestingly another study also highlights that there is a significant connection between two genetic variations -511C/T (rs16944) and -31T/C (rs1143627) based on an analysis of linkage disequilibrium, indicating that the -511C/T variation is almost always inherited together with the-31T/C variation. Essentially, these genetic changes are closely linked, and when one is present, the other is likely to be present as well [39]. In several combined genotypic studies, the likelihood of depression declined in cases with the T/C-C/T genotype of rs1799964-rs1143627 [40] and the G/G—C/C combined genotype of rs1800629-rs1143627 [41].

According to the present study, in the case of rs1143627, it was found that the CT heterozygote genotype tended to increase significantly 2.22 times risk for the development of MDD when compared with the CC common homozygote genotype. But in the case of the TT rare homozygote, it showed a 4.44 times greater risk for MDD when compared with the CC common homozygote genotype and the association was statistically insignificant. In the recessive model and over-dominant model, it tended to increase by 3.58 times and 2.15 times greater risk for the development of MDD respectively but in the recessive model it was insignificant and in the case of over dominant model the risk was statistically significant (p-value = 0.036). On the other hand, in the dominant model, the allele association was at a higher risk of significant correlation between the genotype carriers (OR = 2.35, p-value = 0.019).

One study reported that polymorphisms of rs1143627 AA and rs16944 GG genotypes of IL-1β in recurrent depression [37]. The rs16944 (511C/T) polymorphism in the IL-1β gene has been linked to depressive symptoms and responses to antidepressant therapy [42]. Studies suggest that individuals with the C allele may experience more severe depressive symptoms and higher IL-1β expression, indicating a potential genetic risk factor for major depression. However, the exact mechanism by which rs16944 affects IL-1β expression remains unclear, with inconsistent results across studies. Research also suggests that the impact of rs16944 may be influenced by interactions with other polymorphisms, such as 31T [27]. Further investigation is required to fully understand the relationship between rs16944 and IL-1β expression. Considering polymorphism of IL-1β at rs16944 position, it was evident that the TC heterozygote genotype had a 1.91-fold higher risk of developing MDD compared to TT common homozygote genotype and there was close to significant association between them. The CC genotype was shown to be related to an 8.20-fold higher risk in this investigation. However, no significant correlation was found between the CC and TT genotypes. In the dominant model, individuals with a TC+CC genotype showed a slightly increased possibility of developing MDD (2.06-fold). There was a statistically significant connection between the two variables. The risk of developing MDD was 6.57 times higher in the CC genotype in the recessive model. The odds ratio for CC compared to TT+TC was 6.57, however, the connection did not achieve statistical significance. The TC allele was shown to have an over-dominant model-based 1.79-fold increased risk of having MDD. Though it displayed a higher risk than in TT+CC genotype carriers, it was statistically insignificant.

However, besides the potential outcomes of this present study, some limitations should be addressed. Firstly, the number of MDD patients and controls included in the study is relatively low. This small sample size of present study might limit the generalization of the findings. Also, only known SNP was selected from a public database without novel SNP. Socio- demographic characteristics of both case and HCs are not statistically significant, so they did not affect the association study. The present study found a statistically significant association of some genotypic distribution between of IL-1β (rs1143627 and rs16944) gene and MDD patients which indicates that the IL-1β gene is a potential biological marker for developing MDD in the Bangladeshi population. The result of both the C and T alleles might also be a higher risk factor for IL-1β (rs1143627 and rs16944) genes as only a few genotypic distributions show significant association, further studies will be required for a more significant result.

## 5. Conclusion

According to our knowledge, this is the first study conducted among the Bangladeshi population to explore genetic associations with MDD risks. The present study has investigated the association between IL-1β rs16944 and rs1143627 polymorphisms and MDD susceptibility among the Bangladeshi population. These findings provide evidence for further investigation of the genetics associated with depression. Therefore, we recommend further large-scale studies to explore and confirm this correlation between the genetic polymorphism of the IL-1β gene and MDD patients.

## Supporting information

S1 Checklist STROBE-checklist-v4-combined-PlosMedicine. (DOCX)
